# Autophagy extends lifespan via vacuolar acidification

**DOI:** 10.15698/mic2014.05.147

**Published:** 2014-05-05

**Authors:** Christoph Ruckenstuhl, Christine Netzberger, Iryna Entfellner, Didac Carmona-Gutierrez, Thomas Kickenweiz, Slaven Stekovic, Christina Gleixner, Christian Schmid, Lisa Klug, Ivan Hajnal, Alice G. Sorgo, Tobias Eisenberg, Sabrina Büttner, Guillermo Marin͂o, Rafael Koziel, Christoph Magnes, Frank Sinner, Thomas R. Pieber, Pidder Jansen-Dürr, Kai-Uwe Fröhlich, Guido Kroemer, Frank Madeo

**Affiliations:** 1 Institute for Molecular Biosciences, University of Graz, 8010 Graz, Austria.; 2 INSERM, U848, F-94805 Villejuif, France.; 3 Institut Gustave Roussy, F-94805 Villejuif, France.; 4 Université Paris Sud, Paris 11, F-94805 Villejuif, France.; 5 Institute for Biomedical Aging Research (IBA), Austrian Academy of Sciences, 6020 Innsbruck, Austria.; 6 HEALTH-Institute for Biomedicine and Health Sciences, Joanneum Research Forschungsgesellschaft m.b.H., 8010 Graz, Austria.; 7 Division of Endocrinology and Metabolism, Department of Internal Medicine, Medical University of Graz, 8036 Graz, Austria.; 8 Metabolomics Platform, Institut Gustave Roussy, F-94805 Villejuif, France.; 9 Centre de Recherche des Cordeliers, F-75005 Paris, France.; 10 Pôle de Biologie, Hôpital Européen Georges Pompidou, AP-HP, F-75908 Paris, France.; 11 Université Paris Descartes, Paris 5, F-75270 Paris, France.

**Keywords:** autophagy, methionine restriction, longevity, chronological lifespan, dietary restriction, vacuole, lysosome, acidification

## Abstract

Methionine restriction (MetR) is one of the rare regimes that prolongs lifespan across species barriers. Using a yeast model, we recently demonstrated that this lifespan extension is promoted by autophagy, which in turn requires vacuolar acidification. Our study is the first to place autophagy as one of the major players required for MetR-mediated longevity. In addition, our work identifies vacuolar acidification as a key downstream element of autophagy induction under MetR, and possibly after rapamycin treatment. Unlike other amino acids, methionine plays pleiotropic roles in many metabolism-relevant pathways. For instance, methionine (i) is the N-terminal amino acid of every newly translated protein; (ii) acts as the central donor of methyl groups through S-adenosyl methionine (SAM) during methylation reactions of proteins, DNA or RNA; and (iii) provides the sulfhydryl groups for FeS-cluster formation and redox detoxification via transsulfuration to cysteine. Intriguingly, MetR causes lifespan extension, both in yeast and in rodents. We could show that in *Saccharomyces cerevisiae*, chronological lifespan (CLS) is increased in two specific methionine-auxotrophic strains (namely Δ*met2* and Δ*met15*).

 In view of the fact that macroautophagy (hereafter described as autophagy) constitutes (one of) the major anti-aging pathway(s), we evaluated the hypothesis that methionine could act as an important and potent autophagic regulator. Indeed, we discovered that MetR induces a rapid and long-lasting increase in autophagic flux. Importantly, deletions of genes that are essential for autophagy (such as *atg5*, *atg7* or *atg8*) largely abolished the MetR-mediated improvement of CLS, demonstrating that autophagy is required for MetR-mediated anti-aging effects. In line with this interpretation, MetR-induced autophagy was epistatic to other autophagy-inducing regimes such as pharmacological and genetic inhibition of the target of rapamycin (TOR) pathway. Both rapamycin treatment and genetic deletion of *tor1* failed to further improve CLS under MetR conditions, yet increased CLS in control conditions, i.e. in a yeast strain prototrophic for methionine.

A recent study performed by another group reported that methionine controls autophagy via SAM-mediated methylation status of protein phosphatase 2A (PP2A). In this pathway, methylated PP2A reportedly dephosphorylates Npr2p, a crucial component of a complex that regulates non-nitrogen-starvation-induced autophagy. Future studies will be needed to clarify whether this constitutes the (sole) mechanism(s) of MetR-mediated autophagy induction during chronological aging.

Next, we addressed the potential downstream consequences of enhanced autophagy. For this, we assessed the influence of MetR on vacuolar acidity, driven by the fact that this parameter has been recently implicated in replicative aging of yeast cells, as well as by the consideration that the vacuole is the yeast equivalent of the lysosome and hence constitutes the final target of the autophagic process. We observed that, in chronological aging, the number of yeast cells displaying an acidic vacuole was inversely related to the availability of methionine. Importantly, the increase in vacuolar acidity observed in cultures grown under MetR conditions (as compared to control conditions, i.e. methionine-prototrophic cultures) manifested before a reduction in the frequency of cell death was detected, suggesting that vacuolar acidity might dictate CLS. In line with this interpretation, deletion of *vph2*, an essential assembly factor of the vacuolar ATPase, reversed the MetR-induced increase of CLS. In addition, overexpression of Vph2p or Vma1p (two components of the vacuolar ATPase that are known to increase vacuolar acidity) extended CLS. Intriguingly, vacuolar acidification is also important for CLS of *Schizosaccharomyces pombe*, a distant relative to baker’s yeast. Thus, similar to baker’s yeast, overexpression of Vma1p also improves CLS of *S. pombe*. Whether autophagy is also involved in *S. pombe* vacuolar acidification remains to be established.

In *S. cerevisiae, *autophagy seems to be the driving force for the improved acidification of the vacuole under MetR: a mutant strain devoid of autophagy showed no improvement in vacuolar acidification upon MetR. Treatment with rapamycin, which is one of the most common pharmacological triggers of autophagy, led to an increased vacuolar and vesicle acidification (Figure 1). Intriguingly, it has been shown that *tor1* deletion increases vacuolar acidity during replicative lifespan, as well.

**Figure 1:**
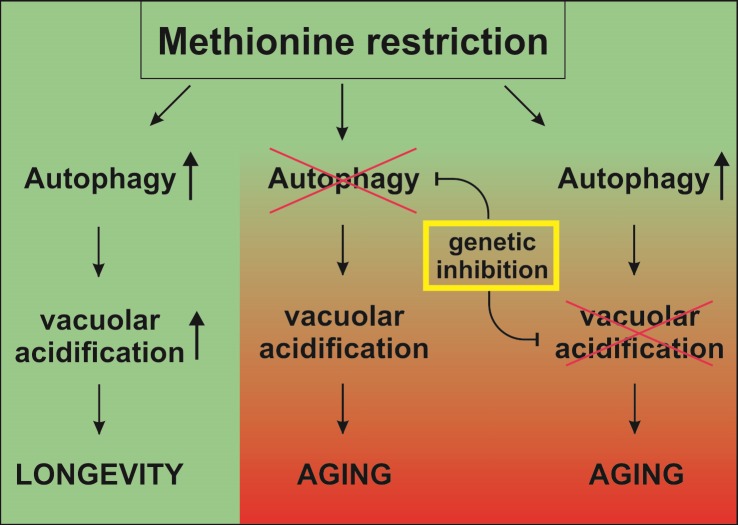
FIGURE 1: Methionine restriction-induced autophagy is causal for longevity-mediating enhancement of vacuolar acidification. Methionine restriction (MetR) specifically induces autophagy during yeast chronological aging, which in turn leads to an increased number of cells bearing acidic vacuoles and ultimately to increased chronological lifespan (CLS). Genetic inhibition of vacuolar acidification by the v-ATPase abrogates the longevity effect of MetR. Also, genetic inhibition of autophagy abrogates the MetR-induced acidification of vacuoles, as well as the MetR-induced extension of CLS.

Lifespan extension by MetR has been conserved from yeast to mammals, suggesting that this phenomenon is of major physiological relevance. Beyond the MetR-induced, autophagy-dependent vacuolar/lysosomal acidification described here, MetR might as well trigger other processes that slow down aging and postpone death. Thus, MetR may trigger an efficient arrest in the G0 phase of the cell cycle (as this has been observed in *S. cerevisiae*) and affect the abundance or proteins from the mitochondrial electron transport chain (as this has been reported for mice). Given the complexity of the aging process, more work is needed to evaluate the possible contribution of these MetR-induced processes to the extension of health span and life span in humans.

